# Identifying Gaps and Missed Opportunities for Intravenous Thrombolytic Treatment of Inpatient Stroke

**DOI:** 10.3389/fneur.2020.00134

**Published:** 2020-02-26

**Authors:** Karan Topiwala, Karan Tarasaria, Ilene Staff, Dawn Beland, Erica Schuyler, Amre Nouh

**Affiliations:** ^1^Department of Neurology, University of Connecticut, Farmington, CT, United States; ^2^Department of Research, Hartford Hospital, Hartford, CT, United States; ^3^Department of Neurology, Ayer Neuroscience Institute, Hartford, CT, United States

**Keywords:** in-hospital stroke, IV-thrombolytic, stroke mimic, missed treatment, quality improvement

## Abstract

**Background:** Inpatient stroke-codes (ISC) have traditionally seen low treatment rates with IV-thrombolytic (IVT). The purpose of this study was to identify the predictors of true stroke, prevalent IVT-treatment gap and study the factors associated with such missed treatment opportunities (MTO).

**Methods:** A retrospective chart review identified ISC from March 2017 to March 2018. Clinical, radiographic and demographic data were collected. Primary analysis was performed between stroke vs. non-stroke diagnoses. Dichotomous variables were analyzed using Chi-Square test of proportions and continuous variables with Wilcoxon-Ranked-Sum test. Significant factors were then tested in a multivariate logistic regression model for independence.

**Results:** From 211 ISC, 36% (*n* = 76) had an acute stroke. Hemorrhagic stroke (HS) was present in 5.7% (*n* = 12). Of the remaining 199, 44% (*n* = 87) were IVT-eligible but only 3.4% (*n* = 3) were treated. Of the remaining 84 IVT-eligible-but-untreated patients, 69(82.1%) were mimics, while 15 (17.9%) had an ischemic stroke (IS), constituting a MTO of 1 in 6 IVT-eligible patients, with National Institutes of Health Stroke Scale (NIHSS) ≤4 being the commonest deterrent. Independent predictors of stroke were ejection fraction (EF) <30% (*p* = 0.030, OR = 3.06), post-operative status (*p* = 0.001, OR = 3.71), visual field-cut (*p* = 0.008, OR = 3.70), and facial droop (*p* = 0.010, OR = 2.59).

**Conclusion:** In our study, one in three ISC were true strokes. IVT treatment rates were low with a MTO of 1 in 6 IVT-eligible patients. The most common reason for not treating was NIHSS ≤4. Knowing predictors of true stroke and the common barriers to IVT treatment can help narrow this treatment gap.

## Introduction

The inpatient population is closely monitored with readily available laboratory information and rapid access to imaging modalities and therapeutic interventions. However, this cohort has frequently been recognized to suffer from delayed recognition, low treatment rates and poor outcomes (1). Several factors related to the patient (old age, co-morbidities, acute illness) remain non-modifiable, but more important are the modifiable factors which are often related to systems-of-care issues (lack of education, in-efficient triaging, delayed physician notification, delayed transport to CT scan) (2). A multitude of mechanisms for the underlying stroke pathophysiology have been identified, with cardioembolism being the most common (3). Treatment rates with IVT for acute strokes presenting to the emergency department range from 2 to 21%, while in the inpatient setting this number drops down to 2.6–11% (4). While recent studies have demonstrated a significant improvement in outcomes by making interventions such as developing new inpatient stroke-code algorithms, educating allied health personnel (5), and early stroke-code activations by nursing staff (6)—the exact rate and burden of MTO from not treating an inpatient-stroke remains unclear. In this study we aim to identify the actual MTO rate within this population. Further, we study the reasons for under-treatment and identify predictors of inpatient stroke, which may aid in narrowing this treatment gap.

## Methods

**Figure 1:**
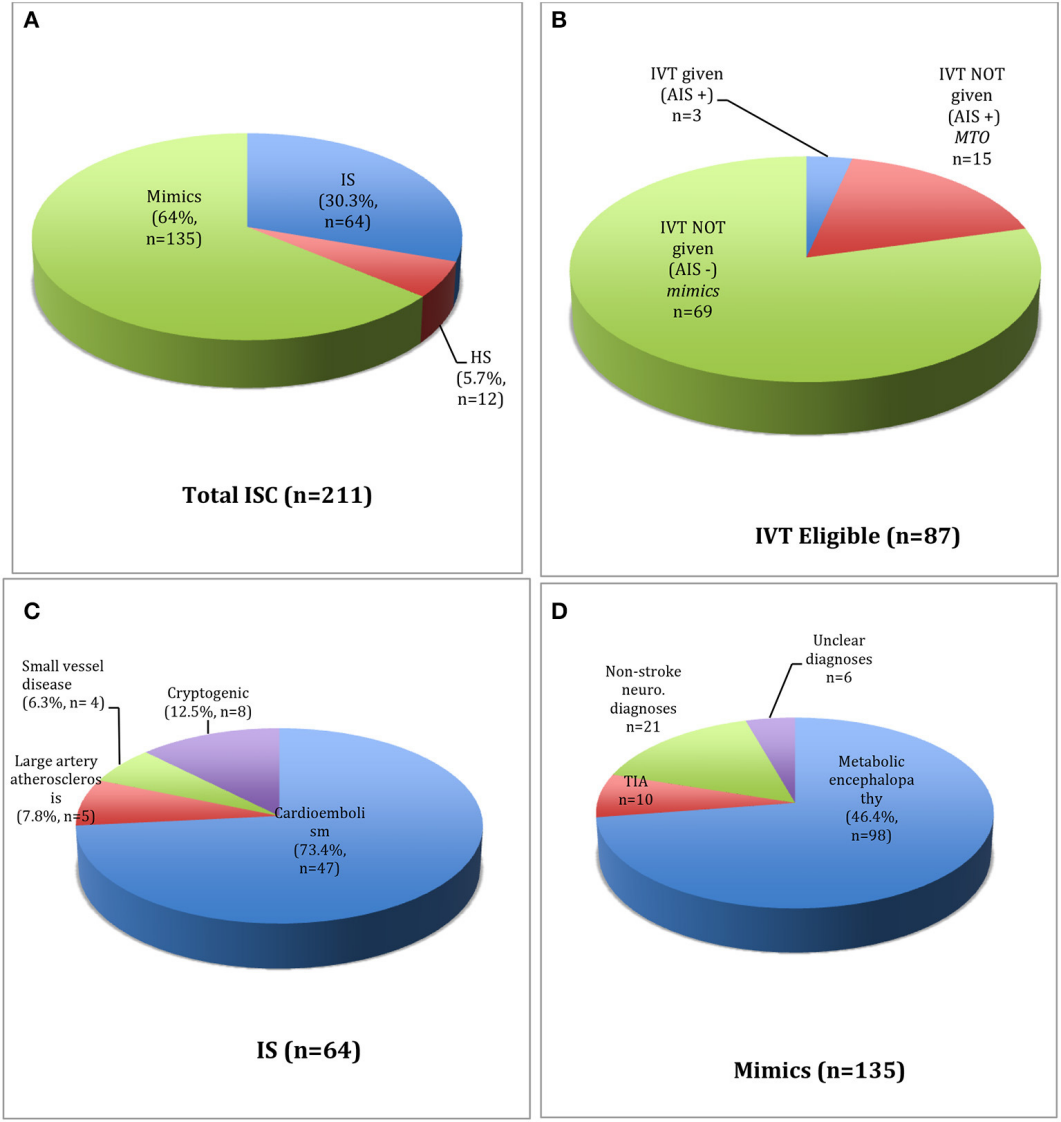
Primary **(A)** and secondary **(B)** study cohorts. Breakdown of IS cases **(C)** and stroke-mimics **(D)** within the primary study cohort.

**Figure 2:**
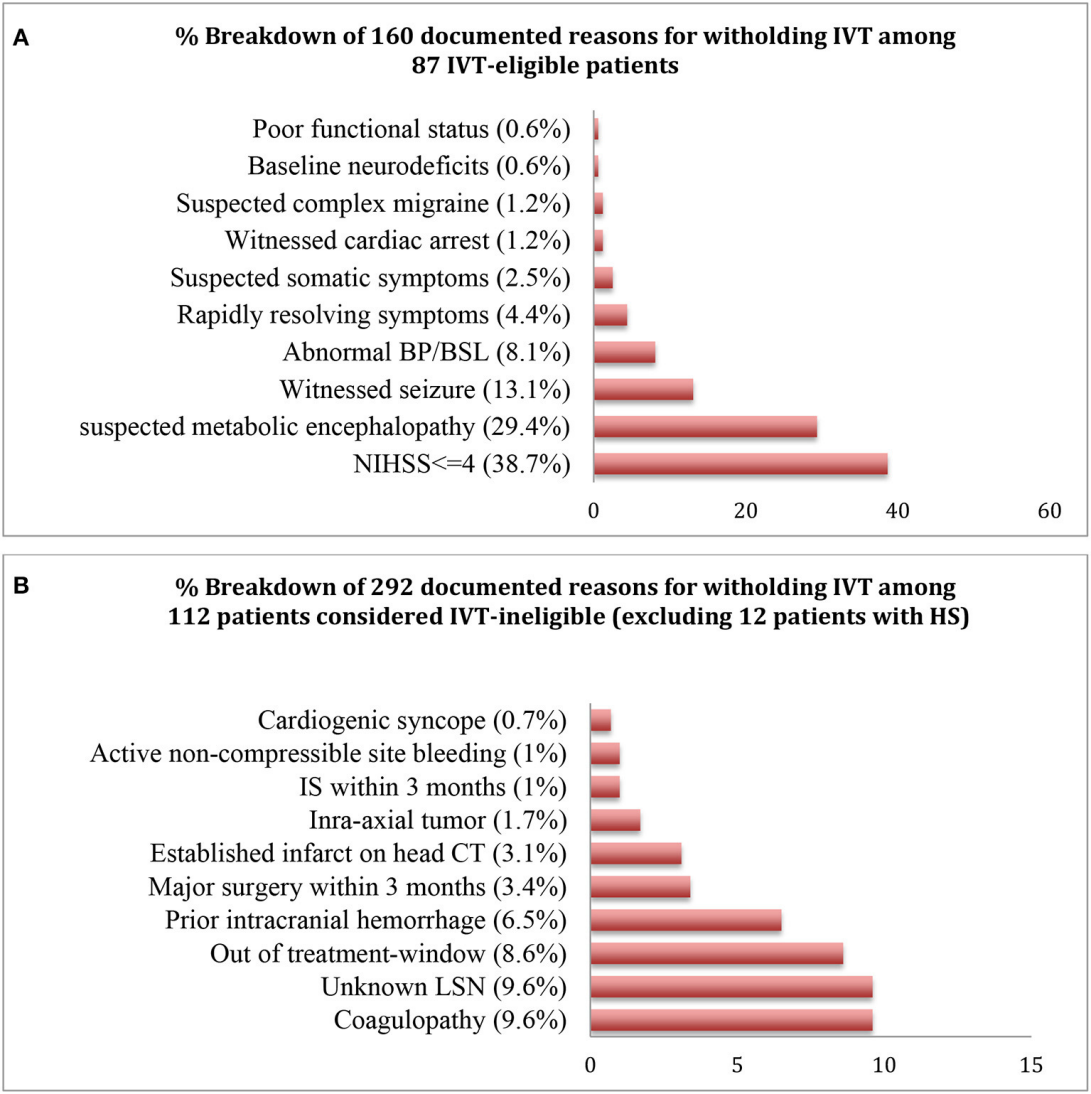
Percentage distributions of the documented reasons for withholding IVT in the IVT-eligible **(A)** and ineligible **(B)** cohorts.

The hospital institutional review board reviewed and approved this study. We performed a retrospective review of prospectively collected data, of hospitalized patients at a Joint Commission certified Comprehensive Stroke Center, from March 2017 to March 2018. Clinical, radiographic and demographic patient data were collected. The primary study cohort included all 211 patients on whom an ISC was called. Primary analysis was performed between stroke vs. non-stroke final diagnoses, on the primary study cohort. Stroke was defined according to the American Heart Association/American Stroke Association guidelines (AHA/ASA) for a new global neurological deficit based on a complete neurological examination by our neurology team as well as corresponding neuroimaging confirming stroke (7). An ISC was called when any member of a patient's care-team (nurse, physical therapist, resident, etc.) noted any new neurological deficit. A neurological deficit was defined as any loss of motor skill, sensory modality, or any change in language or mental status exam. A neurology resident and vascular-neurology trained attending physician team ran all ISC, with a nurse with stroke training also being one of the first responders. All patients underwent initial neuroimaging with non-contrast head CT at the time of ISC. CT angiography (CTA) of the head and neck was the preferred modality of vascular imaging. Subsequent management decisions including administration of IVT, obtaining a CT perfusion (CTP) study, and performing mechanical thrombectomy (MT) were made in accordance with the AHA/ASA standards for stroke management (7). Recanalization after MT was graded using the thrombolysis in cerebral infarction (TICI) score (8). Magnetic resonance imaging (MRI) of the brain was obtained whenever possible. Strokes were categorized as IS or HS. IS was further classified according to the TOAST criteria into cardioembolism, large-artery atherosclerosis, small-vessel disease, cryptogenic, and stroke of other determined etiology. Non-stroke neurological diagnoses were labeled according to the final diagnosis achieved by the neurology consult team (Figure 1). The primary aim of our study was to identify clinical and demographic risk factors, which can significantly differentiate between true strokes and non-stroke events [both, neurologic such as posterior reversible encephalopathy syndrome (PRES), transient ischemic attack (TIA) or seizures as well as systemic, such as sepsis] among hospitalized inpatients presenting with symptoms sufficient to currently result in a stroke code. The secondary aim of the study was to identify the actual treatment rate, treatment gap and barriers to treatment among IVT-eligible patients. Patients were labeled as being IVT-eligible when their bedside examination was consistent with a stroke syndrome, and they were within the treatment window, without any absolute contraindications for IVT therapy. MTO was defined as IVT-eligible patients who did not receive IVT based on one or more reasons (Figure 2), and who subsequently were found to have had an acute IS on follow-up neuroimaging. We collected the following relevant clinical and laboratory variables in all patients including age, sex, diabetes mellitus (DM), EF < 30%, atrial fibrillation (A. fib), symptom onset within 6 h of hemodialysis (HD), sedative medication use, anticoagulant medication use, antiplatelet medication use, admission diagnosis, ward service, perioperative status, initial National Institutes of Health Stroke Scale (NIHSS), last seen normal (LSN), loss of consciousness (LOC), altered mental status (AMS), visual field-cut, aphasia, dysarthria, neglect, unilateral arm or leg weakness, facial droop, sensory symptoms, ataxia, dizziness, blood pressure (BP, <180 mmHg OR ≥180 mmHg) at the time of the stroke code, blood sugar level (BSL, <400 mg/dL OR ≥400 mg/dL) at the time of the stroke code and witnessed seizure activity.

Dichotomous and continuous variables were analyzed using Chi-Square test of proportions and Wilcoxon Ranked Sum test, respectively. Primary analysis was performed between acute-stroke vs. non-stroke diagnoses on primary study cohort. Factors found significant on univariate analysis were then subjected to a multivariate logistic-regression-analysis to study their independence. Further, sensitivity (Se.), specificity (Sp.), negative predictive value (NPV), positive predictive value (PPV) and diagnostic accuracy (DA) for all factors found significant on univariate analysis, were also calculated. A secondary analysis was also performed comparing stroke vs. non-stroke cases, within a secondary cohort comprised only of IVT-eligible patients Statistical significance was defined as 2-tailed *P* < 0.05. All analyses were performed using SPSS v.21 (IBM corporation, Armonk, NY, US).

## Results

A total of 211 ISC were called between March 2017 and March 2018. Cardiovascular symptoms (40.8%) were the most common reason for hospitalization, followed by neurologic (14.2%) and infectious (12.8%) causes (Table 1). From 211 ISC, 76 patients (36%) had an acute stroke [IS, 30.3% (*n* = 64); HS, 5.7% (*n* = 12)] (Figure 1). Of the 64 IS, 54 (84.4%) were confirmed on MRI, while those with incompatible hardware or hemodynamic instability were confirmed to have a new hypodensity on follow-up head CT and a new neurologic deficit on exam. TOAST criteria were used to further classify the 64 IS into cardioembolic (*n* = 47, 73.4%); large-artery atherosclerosis (*n* = 5, 7.8%); small-vessel disease (*n* = 4, 6.3%) and cryptogenic (*n* = 8, 12.5%). Eleven of the 64 IS (17.2%) had a LVO, with 9 occurring in the anterior circulation [internal carotid artery, *n* = 1; middle cerebral artery (MCA), *n* = 8 (M1 MCA = 4, M2 MCA = 2, M3 MCA = 2)] and 2 in the posterior circulation [vertebral artery, *n* = 1; basilar artery, *n* = 1). From 211 codes, 46.4% (*n* = 98) were diagnosed with metabolic encephalopathy, 4.7% (*n* = 10) with TIA, 10% (*n* = 21) with a non-stroke neurological diagnosis and 2.9% (*n* = 6) with an unclear diagnosis. Non-stroke neurological diagnoses included metastatic brain disease (*n* = 7), PRES (*n* = 3), subdural hemorrhage (*n* = 6), intracranial hypotension from ventriculo-peritoneal shunt malfunction (*n* = 1), cryptococcal meningo-encephalitis (*n* = 1), left fronto-parietal non-specific white-matter disease (*n* = 1), meningioma with vasogenic edema and mass effect (*n* = 1), suprasellar mass (*n* = 1). From 211 codes, 10% (*n* = 21) had a witnessed seizure, only 1 of which had an acute IS on MRI brain.

**Table 1:** Symptoms leading to hospitalization.

**No**.	**Admission symptoms**	**Number (*n*)**	**(%)**
1	Cardiovascular	86	40.8
2	Neurological	30	14.2
3	Infectious	27	12.8
4	Gastrointestinal	20	9.5
5	Other	16	7.6
6	Trauma	14	6.6
7	Pulmonary	9	4.3
8	Genitourinary	7	3.3
9	Hematologic	2	0.9
	Total	211	100

On univariate analysis (Table 2), patients with acute stroke were older with systolic heart failure, recent surgery and found to have a facial droop, gaze deviation, visual field-cut or neglect on exam. However, on logistic regression, factors that independently predicted stroke were EF<30% (*p* = 0.030, OR = 3.06), post-operative status (*p* = 0.001, OR = 3.71), visual field-cut (*p* = 0.008, OR = 3.70) and facial droop (*p* = 0.010, OR = 2.59). Among them, being post-operative was the most sensitive (46%), while having an EF<30% was the most specific (94%) with the highest diagnostic accuracy (68%). Sedative use (*p* = 0.049, OR = 0.40) and seizure at onset (*p* = 0.015, OR = 0.07) were inversely predictive of stroke (Table 3). A secondary analysis comparing patients with acute stroke (*n* = 18) to stroke mimics (*n* = 69) within the secondary cohort of IVT-eligible patients (*n* = 87) was also performed. As was found in the full cohort analysis, older age (*p* = 0.006), atrial fibrillation (*p* = 0.019, OR = 4.2 [1.31–13.78]), and facial droop (*p* = 0.049, OR = 3.35 [1.07–10.55]) were significant predictors of acute stroke on univariate analysis. Of the other factors found in the full study, ejection fraction and visual field cut showed similar risk levels in this subgroup but the association did not meet the threshold for statistical significance; it should be noted that with a smaller sample, there is lower statistical power. As was done before, those factors found significant were then entered into a multivariate logistic regression model to test for independence. Only age (*p* = 0.038, OR = 1.05 [1.00–1.10]) retained its significance but this finding must be interpreted carefully as the sample size in the IVT-eligible cohort was low with a lower incidence of stroke (18/87 = 20.7%) compared to primary study cohort (76/211 = 36%). Overall the findings for the primary and secondary analysis are comparable, reflecting a consistency of risk factors regardless of IVT-eligibility.

**Table 2:** Univariate analysis across patient characteristics.

**Parameter**	**Acute stroke** **(*n* = 76)**	**Stroke mimic** **(*n* = 135)**	***P*-value (OR, 95% CI)**
Age (Years)	Median: 70 (28–93)	Median: 69 (19–91)	0.007
Sex (M:F)	33:43	65:70	0.321 (0.75, 0.43–1.32)
**Co-morbidities:** ***n*** **(%)**			
DM	29 (38.16)	54 (40)	0.793 (0.96, 0.52–1.65)
A. fib	30 (39.47)	26 (19.26)	0.001 (2.73, 1.46–5.12)
EF < 30%	16 (21.05)	8 (5.92)	0.001 (4.23, 1.72–10.44)
**Clinical scenario:** ***n*** **(%)**			
Within 6 h. of HD	1 (1.31)	7 (5.18)	0.263 (0.24, 0.29–2.02)
Sedation	11 (14.47)	39 (28.89)	0.018 (0.42, 0.19–0.87)
Anti-coagulation	27 (35.53)	27 (20)	0.013 (2.20, 1.17–4.14)
Anti-platelet	32 (42.11)	49 (36.29)	0.405 (1.28, 0.72–2.27)
Post-operative	35 (46.05)	39 (28.89)	0.012 (2.10, 1.17–3.77)
Elevated level of care	29 (38.16)	41 (30.37)	0.249 (1.42, 0.78–2.55)
Witnessed seizure	1 (1.31)	20 (14.81)	0.002 (0.77, 0.01–0.58)
**Symptoms:** ***n*** **(%)**			
AMS	44 (57.89)	90 (66.67)	0.204 (0.68, 0.38–1.23)
LOC	10 (13.16)	25 (18.52)	0.315 (0.67, 0.30–1.47)
Field-cut	17 (22.37)	11 (8.15)	0.003 (3.25, 1.43–7.37)
Gaze deviation	6 (7.89)	11 (8.15)	0.984 (0.96, 0.34–2.72)
Aphasia	38 (50)	61 (45.18)	0.501 (1.21, 0.69–2.13)
Neglect	10 (13.16)	5 (3.70)	0.010 (3.94, 1.29–11.9)
Dysarthria	39 (51.31)	64 (47.41)	0.586 (1.17, 0.66–2.05)
Motor symptoms	45 (59.21)	79 (58.51)	0.992 (1.03, 0.58–1.82)
Sensory symptoms	33 (43.42)	41 (30.37)	0.057 (1.76, 0.98–3.15)
Ataxia	5 (6.58)	2 (1.48)	0.101 (4.68, 0.88–24.7)
Facial droop	32 (42.11)	28 (20.74)	0.001 (2.78, 1.50–5.15)
Dizziness	5 (6.58)	8 (5.92)	1.000 (1.12, 0.35–3.56)
NIHSS	Median: 6 (0–33)	Median: 4 (0–34)	0.062
LSN	Median: 60 (1–1,140)	Median: 45 (1–1,380)	0.092

*AMS, altered mental status; A. fib, atrial fibrillation; CI, confidence interval; DM, diabetes mellitus; EF, ejection fraction; F, female; HD, hemodialysis; M, male; LOC, loss of consciousness; LSN, last seen normal; OR, odds ratio*.

**Table 3:** Multivariate logistic regression.

**Parameter**	***P*-value (OR, 95% CI)**	**Se., Sp. (%)**	**PPV, NPV (%)**	**DA (%)**
Age (Years)	0.143 (1.02, 0.99–1.05)	65.8, 65.6	45.5, 74.3	59.2
A. fib	0.059 (2.12, 0.97–4.64)	39.5, 80.7	53.6, 70.3	65.9
EF < 30%	0.030 (3.06, 1.11–8.44)	21.1, 94.1	66.7, 67.9	67.8
Sedation	0.049 (0.40, 0.16–0.99)	14.5, 71.1	22, 59.6	50.7
Anti-coagulation	0.654 (1.20, 0.54–2.69)	35.5, 80	50, 68.8	63.9
Post-operative	0.001 (3.71, 1.77–7.78)	46.1, 71.1	47.3, 70.1	62.1
Witnessed seizure	0.015 (0.07, 0.01–0.58)	1.3, 85.2	4.8, 60.5	54.9
Field-cut	0.008 (3.70, 1.40–9.71)	22.4, 91.8	60.7, 67.8	66.8
Neglect	NA^*^	NA	NA	NA
Facial droop	0.010 (2.59, 1.25–5.39)	42.1, 79.3	53.3, 70.9	65.9

*CI, confidence interval; DA, Diagnostic accuracy; NA, not applicable; NPV, negative predictive value; OR, odds ratio; PPV, positive predictive value; Se., sensitivity; Sp., specificity*.

* *Neglect was removed from multivariate analysis secondary to its low frequency*.

Only 3 patients (4.7% of 64 IS *or* 3.4% of 87 IVT-eligible *or* 1.4% of 211 codes) received IVT, all of whom had a confirmed stroke on MRI. Of 84 IVT eligible-but-untreated patients, 160 reasons were noted for not treating, with 44% (*n* = 37) having >1 reason to hold treatment (Figure 2). NIHSS ≤4 was the commonest reasons for withholding treatment among the 87 patients in the IVT-eligible cohort. Notably, from 64 IS cases, 28 patients (43.7%) had a NIHSS ≤4 (embolic, *n* = 25 which included 2 patients with a LVO; lacunar, *n* = 2; watershed, *n* = 1). Five of the 11 LVO's met criteria for MT, achieving successful recanalization (TICI 2b/III) in all cases. Two of 5 MT cases were in the late (6–24 h) window and of the other three, none were IVT-eligible secondary to a coagulopathy (*n* = 2) and ischemic stroke within past 3-months (*n* = 1). Reasons for deferring MT in 6 patients with LVO were: non-favorable CTP study (*n* = 2), clot location deemed un-amenable for MT by the treatment team (*n* = 3), and rapidly resolving symptoms with NIHSS <6 (*n* = 1). Finally, within the 84 patients in the eligible-but-untreated cohort, 82.1% (*n* = 69) were stroke mimics while 17.9% (*n* = 15) had IS, constituting a MTO of 1 in 6 patients.

## Discussion

Inpatient stroke accounts for 7–15% of all acute cerebrovascular events (2). The underlying mechanism is predominantly embolic, especially cardioembolism (73.4% in our cohort). In contrast, only 4 patients had a lacunar stroke with only one (1.6% of the 64 IS) developing clumsy-hand-dysarthria syndrome with MRI demonstrating an infarct in the paramedian pons. Often this population has comorbidities such as sepsis/leukocytosis and anemia, which have been shown to independently increase the risk for thrombogenesis (9, 10). A majority of the inpatient stroke population is warded on cardiology related floors, with one retrospective series of 111 inpatient-strokes in Korea showing that those on cardiology-related departments had a 10-fold higher frequency of stroke (4). In our cohort, patients who were immediately post-operative had significantly higher odds (3.71) of having an acute IS. This is again similar to prior reports, with one study observing that 55% of the procedure associated strokes developed within 1 day, with the remaining cases occurring within 7 days of the procedure (4). The mechanism of delayed procedure associated stroke has also been thought to be related to atrial fibrillation with one study observing that 19% of patients who were post-CABG developed atrial fibrillation (4).

In our cohort A. fib though found to be significant on univariate analysis, was not independently predictive of stroke on logistic regression analysis. However, systolic heart failure with an EF < 30% (*p* = 0.030, OR = 3.06) predicted true stroke with the highest specificity (94%) and diagnostic accuracy (68%). Among other independent predictors were presence of a facial droop (*p* = 0.010, OR = 2.59) and a visual field-cut on exam (*p* = 0.008, OR = 3.70). As would be expected, we found that use of sedation (*p* = 0.049, OR = 0.40) and a witnessed seizure at onset (*p* = 0.015, OR = 0.07) were negative independent predictors of the stroke-code being an actual stroke.

Many recent studies have demonstrated a significant improvement in outcomes using interventions such as developing specific inpatient stroke-code algorithms (5), and early activation of stroke alerts by nursing staff (6), but yet the exact burden of missed opportunities from not treating an inpatient-stroke remains unclear. In our study, 87 patients developed a new neurodeficit and did not have an absolute contraindication for thrombolytic therapy, and were within the IVT treatment-window. They were considered tPA-eligible, from whom only 3 patients (3.5%) received IVT. Of the remaining 84 IVT-eligible-but-untreated patients, 15 (17.9%) were not treated and had a new IS on neuroimaging; and 69 (82.1%) were not treated and did not have a new stroke on imaging (stroke mimics). This conferred a MTO of about 1 in 6 IVT-eligible patients who were not treated and were found to have an acute IS on follow-up imaging. Forty-four percent patients (*n* = 37) within the IVT-eligible-but-untreated cohort had >1 reason to hold treatment, with the number of cited reasons totaling up to 160, with the most common deterrent being NIHSS ≤4 (38.7%). IVT treatment in strokes presenting with mild symptoms, often considered to be the case when initial NIHSS is ≤4, has been considered a controversial issue. However, the first double-blinded, randomized controlled trial (PRISMS) assessing IVT vs. aspirin (325 mg) use for mild, non-disabling stroke in 313 patients (median NIHSS, 2) showed no clear benefit of IVT over aspirin, but did demonstrate a higher rate of symptomatic hemorrhage (3.2 vs. 0%) (11). However, only 13.6% cases in this study had a cardioembolic stroke with the predominant etiology being small vessel disease (36.6%), contrary to cardioembolism as the predominant pathophysiology for inpatient IS. The study had other limitations including early termination because of slow recruitment (11). Thus, while use of IVT in patients with mild stroke without disabling symptoms, showed harm without any suggestion of benefit, this has not been conclusively been shown to be the case for inpatient-stroke.

Finally, we highlight the mimic rate of >80% in the IVT-eligible-but-untreated group. Previous reports have documented similarly high mimic rates, with one study comparing inpatient-to-ED strokes revealing that being inpatient was itself an independent predictor of the code being a mimic (12). This must be weighed against potential MTO. Further studies are needed to assess the currently prevalent MTO rates and study IVT for inpatients with low NIHSS.

Limitations of our study include being a single-center retrospective analysis, which introduces the risk of selection bias. The median NIHSS in our cohort (6 in patients with AIS, 4 in stroke-mimics) is lower than the median NIHSS (9) reported previously in a large nationwide inpatient-cohort (13). This could be a result of our low threshold for ISC activation, uncovering a larger inpatient population with minor stroke. At the same time, the NIHSS documentation rate in the above cohort was 56% overall, with a reported selection bias favoring higher NIHSS scores from facilities with low NIHSS documentation rates (14). Finally, our inpatient cohort represents a small sample. Collectively, these factors may limit the external validity of our findings. Despite these limitations, our study highlights IVT utilization by identifying the missed treatment opportunities, while also recognizing the barriers to treatment in the inpatient population. Further studies are needed to assess the currently prevalent MTO rates among inpatients, across different healthcare settings.

## Conclusion

In our study, 1 in 3 ISC were true strokes and IVT treatment rates were low with a MTO of 1 in 6 IVT-eligible patients. The most common reason for not treating was NIHSS ≤4. Knowing predictors of true stroke and the common barriers to IVT treatment can help narrow this treatment gap.

## Data Availability Statement

The datasets generated for this study are available on request to the corresponding author.

## Ethics Statement

The studies involving human participants were reviewed and approved by Hartford Hospital Institutional Review Board. Written informed consent for participation was not required for this study in accordance with the national legislation and the institutional requirements.

## Author Contributions

KTo: study concept and design, data collection and analysis, drafting and revision of manuscript, and full responsibility of data. KTa: data collection, analysis, and manuscript revision. IS and DB: data collection and statistical analysis. ES and AN: study concept and design, critical revision, and review of final manuscript.

### Conflict of Interest

AN, Steering committee and speakers bureau, Genentech. The remaining authors declare that the research was conducted in the absence of any commercial or financial relationships that could be construed as a potential conflict of interest.

## Abbreviations

AMS: altered mental status
AHA/ASA: American Heart Association/American Stroke Association guidelines
A. fib: atrial fibrillation
CI: confidence interval
CT: computed tomography
DA: Diagnostic accuracy
DM: diabetes mellitus
EF: ejection fraction
F: female
HS: hemorrhagic stroke
HD: hemodialysis
ISC: inpatient stroke-code
IS: ischemic stroke
IVT: intravenous-thrombolytic
M: male
LOC: loss of consciousness
LSN: last seen normal
LVO: large-vessel-occlusion
MCA: middle cerebral artery
MRI: magnetic resonance imaging
MTO: missed treatment opportunities
NIHSS: national institutes of health stroke scale
NA: not applicable
NPV: negative predictive value
OR: odds ratio
PPV: positive predictive value
PRES: posterior reversible encephalopathy syndrome
Se.: sensitivity
Sp.: specificity
TIA: transient ischemic attack.
